# Muscone suppresses gastric cancer via regulation of miRNA‐145

**DOI:** 10.1002/fsn3.2269

**Published:** 2021-07-26

**Authors:** Feng Gao, Shihai Yan, Zheng Sun, Jia Wang

**Affiliations:** ^1^ Department of Clinical Laboratory Jiangsu Province Hospital of Chinese Medicine Affiliated Hospital of Nanjing University of Chinese Medicine Nanjing China

**Keywords:** apoptosis, invasion, migration, miRNA‐145, muscone, proliferation

## Abstract

This study aims to determine the effects and mechanism of action of muscone on the biological activity of the gastric cancer cell lines SGC‐7901 and MGC‐803 (proliferation, apoptosis, invasion, and migration) in vitro. An optimal muscone concentration was determined using MTT and cell apoptosis tests. The SGC‐7901 and MGC‐803 cells were divided into five groups: normal control, muscone, miRNA, muscone + miRNA, and muscone + miRNA inhibitor. Cell proliferation rate, apoptosis rate, cell cycle phase distribution, number of invading cells, and wound healing rate were compared among the five groups using MTT, flow cytometry, transwell, and wound healing assays. Relative expression levels of the proteins PI3K, AKT, P21, c‐Myc, MMP‐2, and MMP‐9 were measured by Western blot. Compared with the control group, the groups treated with muscone and miRNA showed significantly lower cell proliferation rate, number of invading cells, and wound healing rate (*p* < .05 for all), but significantly higher rates of cell apoptosis rate and numbers of cells in the G1 phase (*p* < .05 for all). These groups also showed significantly lower expression of the proteins PI3K, AKT, c‐Myc, MMP‐2, and MMP‐9 but significantly increased expression of the protein P21 (*p* < .05). Transfecting muscone‐treated SGC‐7901 and MGC‐803 cells with miRNA‐145 inhibitor resulted in a significant recovery of biological activity (*p* < .05). Muscone suppresses the biological activity of SGC‐7901 and MGC‐803 gastric cancer cells in vitro via regulation of miRNA‐145.

## Graphical abstract

Muscone suppressed SGC‐7901 biological activities via regulation of miRNA‐145 in vitro.

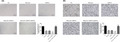

## 1 INTRODUCTION

Muscone is the primary functional component of the natural musk produced by animals such as the musk deer and muskrat. It is usually extracted from the musk sacs or dry exocrine secretions of mature male musk deer (Wang et al., 2020). Some studies have reported that muscone exerts anti‐inflammatory effects (Du et al., 2018; He et al., 2020). However, it remains unclear whether muscone exerts antitumor effects in gastric cancer.

MicroRNAs (miRNAs) are a class of small, single‐stranded, noncoding RNAs that are found widely in eukaryotic cells. miRNAs are 19–25 nucleotides in length and serve important biological functions such as regulating downstream gene transcription by binding to mRNA transcripts of target genes (Hannafon & Ding, 2018). miRNA‐145 is a miRNA that plays a key role in cancer development through its effects on cell proliferation, apoptosis, invasion, and migration (Yu et al., 2017; Zhang et al., 2019; Zhang & Song, 2019). Previous studies have confirmed that 40% of miRNA‐coding genes are located either at fragile sites involved in tumors or near the breakpoints of oncogenes and tumor suppressor genes (Kim et al., 2018; Zhang et al., 2017). These studies have shown that miRNAs play a key role in the progression of malignant tumors, serving a similar function to tumor suppressor genes and oncogenes. Based on these studies, we decided to test the ability of muscone to exert antitumor effects by regulating miRNAs. We first determined the optimal muscone concentration using MTT and cell apoptosis tests. Next, we compared the inhibitory effects of muscone alone, muscone combined with miRNA‐145, and muscone combined with miRNA‐145 inhibitor against gastric carcinoma cells (SGC‐7901 and MGC‐803). Finally, we determined the main affected signal pathway using the western blot (WB) assay.

## 2 MATERIALS AND METHODS

### 2.1 Reagents

Muscone was purchased from Sigma (USA). Flow cytometry was performed using an annexin V‐FITC/PI (annexin V–fluorescein isothiocyanate/propidium iodide) kit (BD, Biosciences), and cells were cultured in RPMI‐1640 medium (Hyclone).

### 2.2 Cell cultures

The SGC‐7901 and MGC‐803 gastric carcinoma cells (ATCC) were cultured in an incubator (5% CO2, 37℃) in RPMI‐1640 medium containing 10% fetal bovine serum (FBS) and 100 U/ml penicillin and streptomycin (Sigma). Cell growth was observed under an inverted microscope, and 0.25% trypsin was used to dissociate the cells for each passage. Cells in the logarithmic growth period were used for the experiment.

### 2.3 Determination of optimal muscone concentration

To determine the optimal muscone concentration for tumor inhibition, MTT and cell apoptosis assays were performed to compare the effects of different concentrations (0 µg/ml, 5 µg/ml, 10 µg/ml, 25 µg/ml, 50 µg/ml, and 100 µg/ml) on SGC‐7901 and MGC‐803 cells.

### 2.4 Cell treatment groups

SGC‐7901 and MGC‐803 cells were divided into five groups: the normal control (NC) group remained untreated; the muscone group was treated with the optimal concentration of muscone; the miRNA group was transfected with miRNA‐145 (GenScript Nanjing Co., Ltd); the muscone + miRNA group was treated with the optimal concentration of muscone and transfected with miRNA‐145; and the muscone + miRNA inhibitor group was treated with the optimal concentration of muscone and transfected with miRNA‐145 inhibitor (GenScript Nanjing Co., Ltd.).

### 2.5 MTT assay

SGC‐7901 and MGC‐803 cells were collected for the MTT assay. The cells were incubated for 48 hr, after which MTT solution (20 μl; Sigma) was added to every well, and incubation was continued for 4 hr before being stopped. Next, the supernatant was removed from the wells, and 150 μl DMSO was added to every well. The solution in the wells was mixed for 10 min to fully dissolve the crystals. The absorbance value at 490 nm was measured for each group by enzyme‐linked immunosorbent assay. Cell proliferation in each group was also measured.

### 2.6 Analysis of cell apoptosis and cell cycle phase distribution by flow cytometry

SGC‐7901 and MGC‐803 cells were collected and dissociated with 0.25% trypsin, after which the cells were collected by centrifugation (2000 *g*, 5 min) and washed twice with pre‐cooled PBS. The cells were then resuspended in 400 μl 1 × binding buffer (Mbchem M3036) at a concentration of 1 × 10^6^ cells/ml. Annexin V–FITC (5 µl) was added to the cell suspension, which was then mixed and protected from light for 15 min at 2–8℃. Next, 10 µl PI was added to the mix, which was then protected from light for 5 min at 2–8℃. Finally, cell apoptosis rates and the number of cells at different stages of the cell cycle were measured using flow cytometry performed over 1 hr.

### 2.7 Transwell migration assay

SGC‐7901 and MGC‐803 cells were collected during the logarithmic growth period, dissociated with 0.25% trypsin, and suspended in RPMI‐1640 medium. The cell concentration of the suspension was adjusted to 5 × 10^4^ cells/100 μl. Each well in a 24‐well plate was filled with 600 μl RPMI‐1640 complete medium containing 15% FBS and 100 μl cell suspension containing 5 × 10^4^ SGC‐7901 and MGC‐803 cells, and 10 μl 1% BSA was added to the upper chamber to adjust the BSA concentration to 0.1% and maintain the osmotic pressure in the upper chamber. SGC‐7901 and MGC‐803 cells were divided into treatment groups as previously described and cultured in a 5% CO_2_ incubator for 15 hr. Thereafter, the chamber was removed, and the cells were fixed with 90% EtOH, stained with 0.1% crystal violet solution, observed, and photographed under a microscope to measure their number. The experiment was repeated three times.

### 2.8 Wound healing assay

SGC‐7901 and MGC‐803 cells were collected and covered in a 6‐well plate, divided into treatment groups as previously described, incubated until wells were full, and washed with PBS. The culture medium was replaced with a serum‐free medium. The scratch width was observed after 0, 24, and 48 hr, and photographs were taken.

### 2.9 Western blot assay

SGC‐7901 and MGC‐803 cells from the different treatments were collected. Cells were lysed on ice for 30 min, and 300 μl cell lysate was transferred to a 1.5‐mL Eppendorf tube and centrifuged at 12,000 r/min and 4℃. The supernatant was collected and stored at −20℃ until use. Protein concentration was measured by the bicinchoninic acid method to ensure 40 μg of protein in every gel lane. Gel electrophoresis was performed using 12% polyacrylamide gel (120 V, 90 min), after which the proteins were transferred to PVDF membranes. Membranes were blocked for 2 hr on a table using PBS solution containing freshly prepared 5% skim milk powder. Primary antibodies against PI3K, AKT, P21, c‐Myc, MMP‐2, MMP‐9, and GAPDH (1:500) were added, and the membranes were stored overnight at 4℃. The next day, the membranes were taken out and washed with PBS (15 min × 3 times). Horseradish peroxidase secondary antibodies were added, and the membranes were incubated for 1 hr at room temperature, followed by washing with PBS (10 min × 3 times) and measurement of chemiluminescence (BD Biosciences). The experiment was repeated 3 times.

### 2.10 RT‐qPCR assay

Cells were collected from different groups and subjected to RT‐qPCR to measure the expression of miRNA‐145 and of mRNA transcripts of *PI3K, AKT*, *P21*, *c‐Myc*, *MMP‐2*, and *MMP‐9*. The primer sequences were purchased from Shanghai Shenggong Bioengineering Co., Ltd. The reaction conditions were as follows: first, denaturation for 2 min at 90℃; next, 35 cycles consisting of 30 s at 93℃, 30 s at 60℃, and 30 s at 80℃; finally, 2 min at 80℃. *U6* was used as a reference gene for comparison with miRNA‐145, and *GAPDH* was used as a reference gene for comparison with the protein‐coding genes. The primer sequences are listed in Table 1.

**TABLE 1:** The primer sequence

Gene Name	F:(5'−3')	R:(5'−3')
miRNA−145	CAGTCTTGTCCAGTTTTCCCAG	TATGCTTGTTCTCGTGTCTGTGTC
U6	CCCTGGCACCCAGCAC	GCCGATCCACACGGAGTAC
MMP−9	CCTTCTACGGCCACTACTGT	TCCACCTGGTTCAACTCACT
MMP−2	CCAACTACAACATCTTCCCTC	TCCGTCCTTACCGTCAAA
c‐Myc	CCTGGTGCTCCATGAGGAGA	TCCAGAAGGTGATCCAGAC
P21	GTCCCGCCAAGGTCTAGCTG	GCTGTATATTCAGCATTGTGGG
PI3K	CATCACTTCCTCCTGCTCTAT	CAGTT2GTTGGCAATCTTCTTC
AKT	GGACAACCGCCATCCAGACT	GCCAGGGACACCTCCATCTC
GAPDH	TCTGACTTCAACAGGGACACC	CTGTTGGTGTAGCCAAATTCGT

### 2.11 Statistical analysis

SPSS software (version 22.0) was used to analyze the experimental data. Measurement data are given as mean ± *SD*. Comparisons between multiple groups were performed using one‐way ANOVA with the least significant difference *t* test. Statistical significance was set at *p* < .05.

## 3 RESULTS

### 3.1 Optimal inhibitory concentration of muscone (MTT and cell apoptosis assays)

**FIGURE 1:**
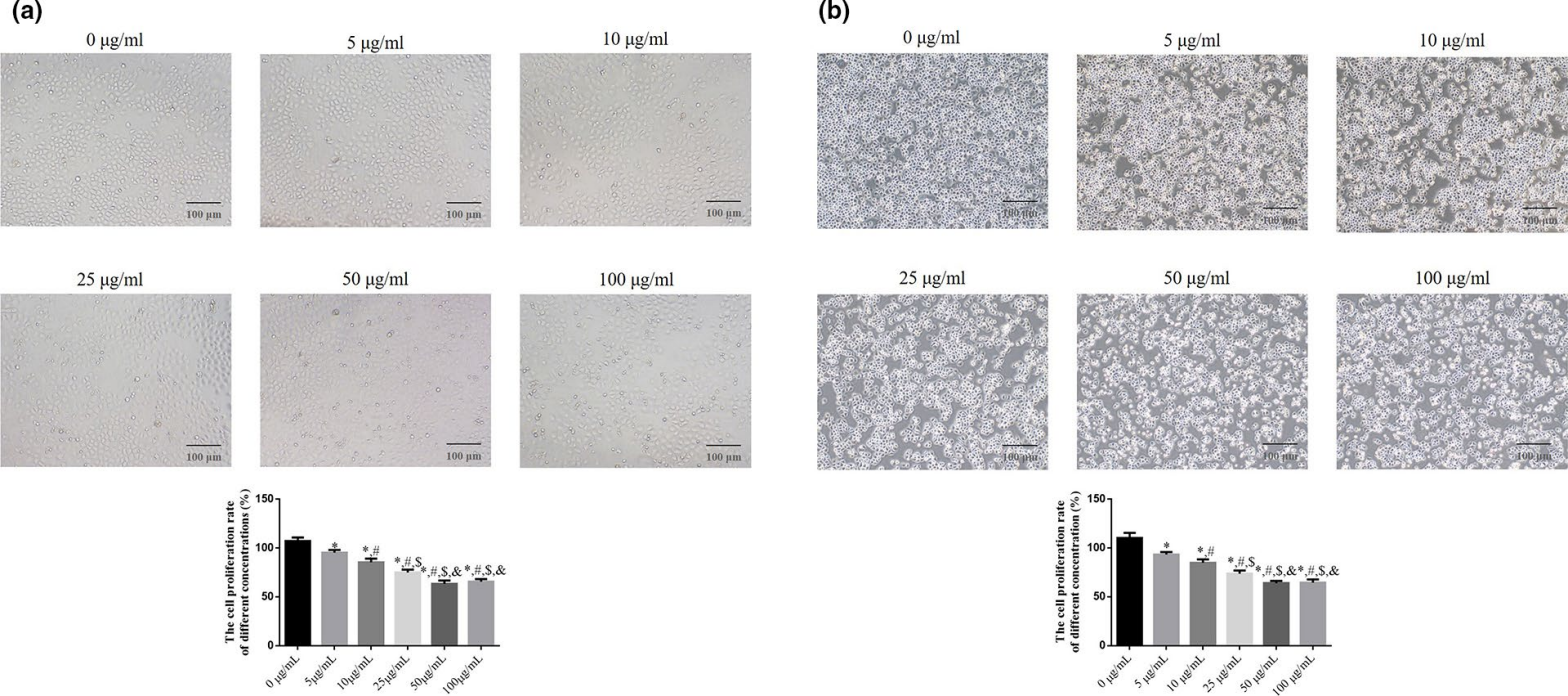
The cell proliferation by MTT assay in difference Muscone concentrations groups (100×). (a) The cell proliferation in SGC‐7901 cell line. (b) The cell proliferation in MGC‐803 cell line. *: *p* < .05, versus. 0 µg/ml; #: *p* < .05, versus. 5 µg/ml; $: *p* < .05, versus. 10 µg/ml; &: *p* < .05, versus. 25 µg/ml

**FIGURE 2:**
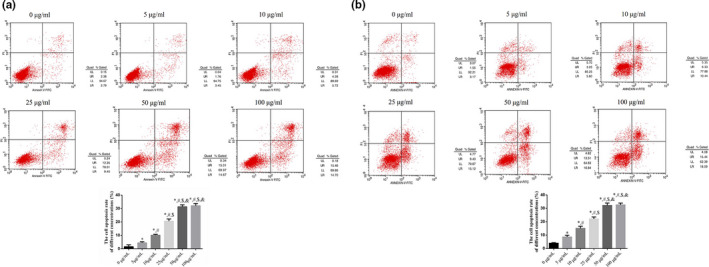
The cell apoptosis rate in difference Muscone concentrations. (a) The cell apoptosis rate in SGC‐7901 cell line. (b) The cell apoptosis rate in MGC‐803 cell line. *: *p* < .05, versus. 0 µg/ml; #: *p* < .05, versus. 5 µg/ml; $: *p* < .05, versus. 10 µg/ml; &: *p* < .05, versus. 25 µg/ml

The MTT and cell apoptosis assays showed that compared with the NC group, cell proliferation in the muscone‐treated groups was significantly suppressed (*p* < .05, Figure 1), and cell apoptosis rate was significantly increased (*p* < .05, Figure 2). There were significant differences among the different concentrations of muscone (5 μg/ml, 10 μg/ml, 25 μg/ml, and 50 μg/ml), with dose‐dependent effects on cell proliferation and apoptosis (*p* < .05; Figures 1 and 2). The only exception was that there were no significant differences in cell proliferation and apoptosis rates between the 50 μg/ml and 100 μg/ml muscone groups (*p* > .05, Figures 1 and 2). Based on these results, 50 μg/ml was selected as the optimal concentration of muscone to inhibit proliferation and stimulate apoptosis in SGC‐7901 and MGC‐803 cells.

### 3.2 Cell proliferation rate (MTT assay)

**FIGURE 3:**
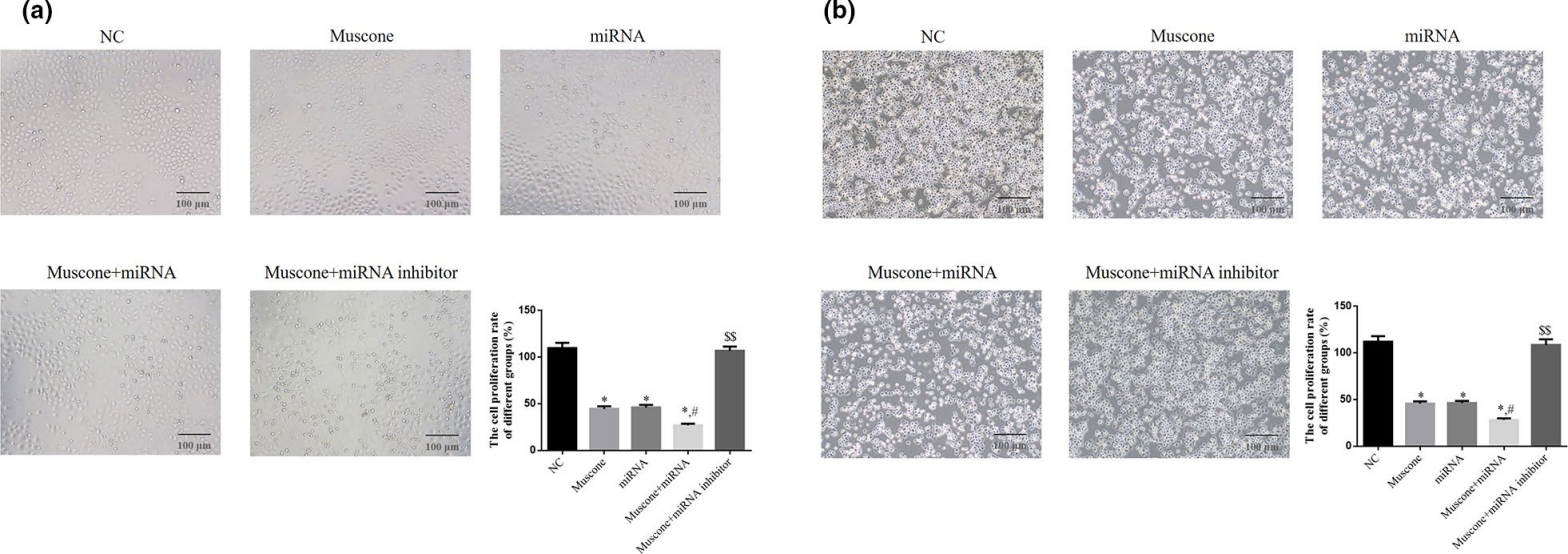
Cell proliferation by MTT assay in difference groups. (a) Cell proliferation in SGC‐7901 cell line. (b) Cell proliferation in MGC‐803 cell line. *: *p* < .05, versus. NC group; #: *p* < .05, versus. Muscone group; $$: *p* < .01, versus. Muscone group

Compared with NC, cell proliferation rates were significantly downregulated in the muscone, miRNA, and muscone + miRNA groups (*p* < .05). Furthermore, the cell proliferation rate of the muscone + miRNA group was significantly lower than that of the muscone group (*p* < .05). However, the proliferation rate of the muscone + miRNA inhibitor group was significantly higher than that of the muscone group (*p* < .05). The respective data are presented in Figure 3.

### 3.3 Cell apoptosis rate (flow cytometry)

**FIGURE 4:**
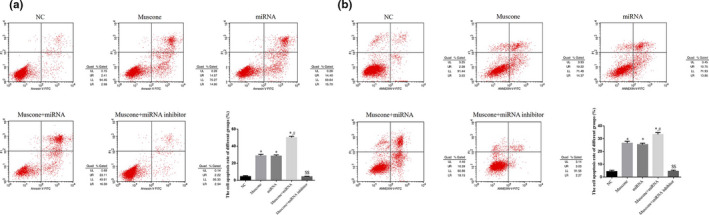
Cell apoptosis rate by flow cytometry in difference groups. (a) The cell apoptosis rate in SGC‐7901 cell line. (b) The cell apoptosis rate in MGC‐803 cell line. *: *p* < .05, versus. NC group; #: *p* < .05, versus. Muscone group; $$: *p* < .01, versus. Muscone group

Flow cytometry showed that the cell apoptosis rates of the muscone, miRNA, and muscone + miRNA groups were significantly upregulated compared with that of the NC group (*p* < .05, respectively), and the cell apoptosis rate of the muscone + miRNA group was significantly greater than that of the muscone group (*p* < .05). However, even with the addition of miRNA‐145, cell apoptosis was significantly suppressed in the muscone + miRNA inhibitor group compared with that in the muscone group (*p* < .05). The respective data are shown in Figure 4.

### 3.4 Cell cycle phase distribution (flow cytometry)

**FIGURE 5:**
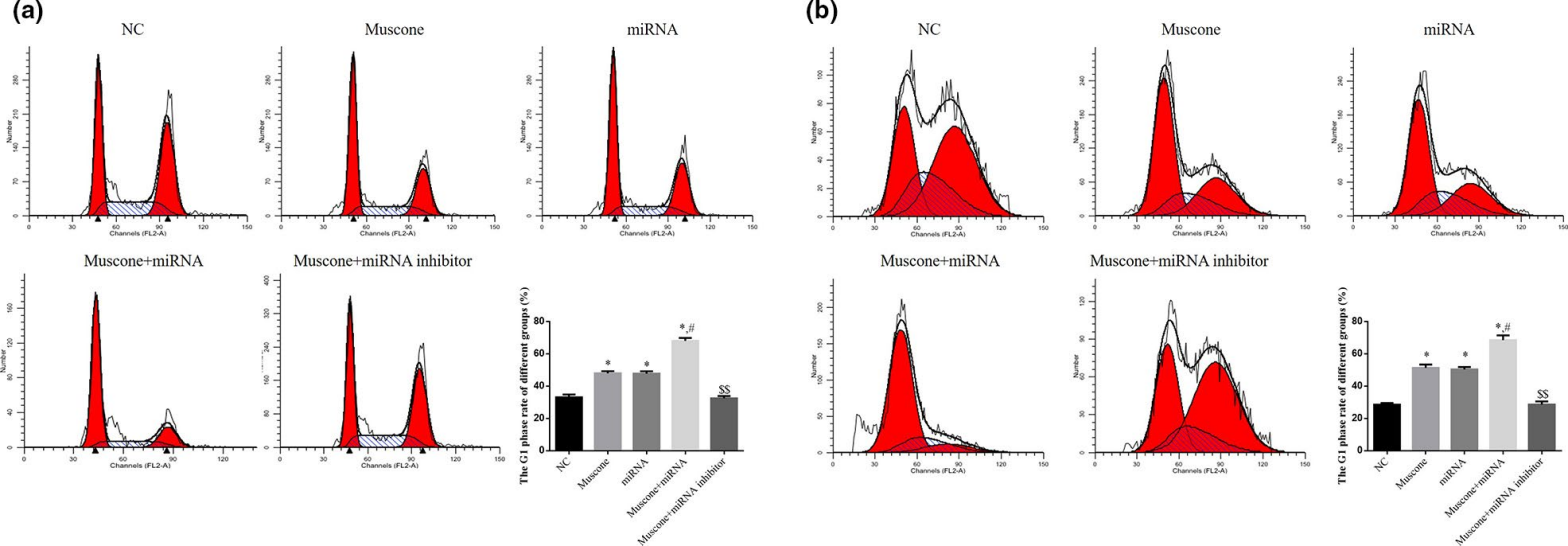
Using flow cytometry to measure cell cycle rates. (a) Cell cycle in SGC‐7901 cell line. (b) Cell cycle in MGC‐803 cell line. *: *p* < .05, versus. NC group; #: *p* < .05, versus. Muscone group; $$: *p* < .01, versus. Muscone group

Based on the flow cytometry assay, we found that the number of cells in G1 phase was significantly increased in the muscone, miRNA, and muscone + miRNA groups compared with the NC group (*p* < .05, respectively). Furthermore, there were significant differences between the muscone + miRNA and muscone groups (*p* < .05). Even with the addition of miRNA‐145, the number of cells in G1 phase rate was significantly lower in the muscone + miRNA inhibitor group than in the muscone group (*p* < .05). The data are shown in Figure 5.

### 3.5 Cell invasion rate (transwell migration assay)

**FIGURE 6:**
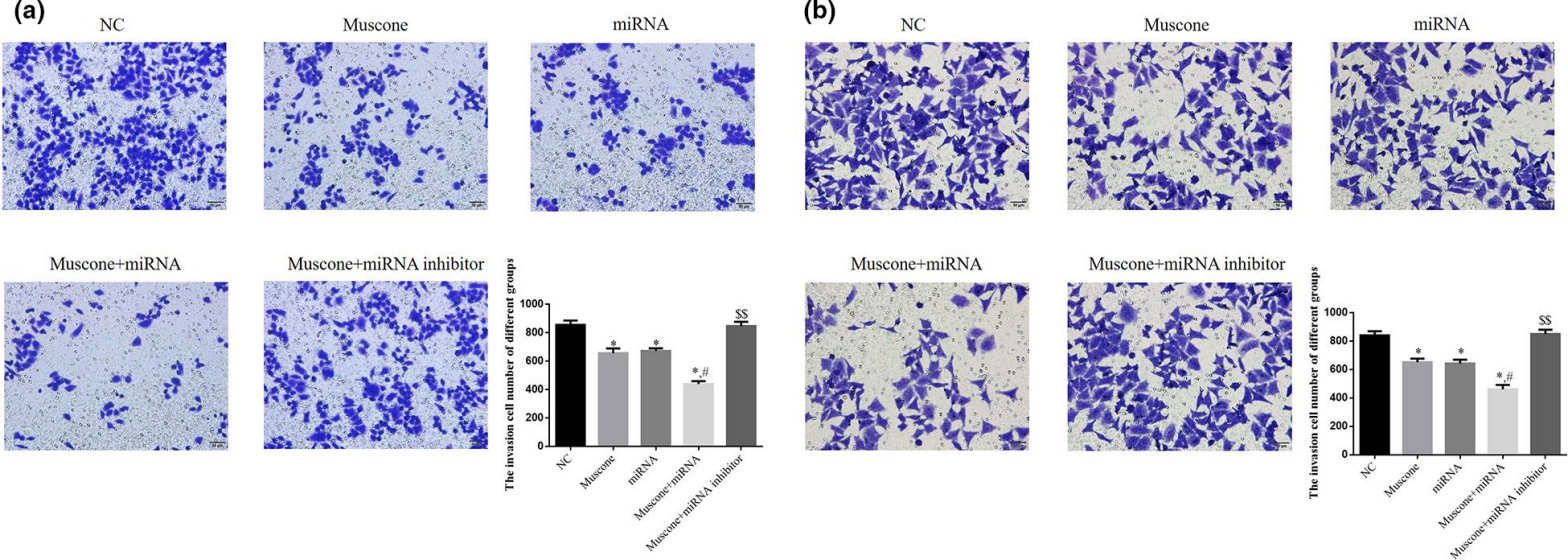
Invasion cell number by transwell assay in difference groups (200×). (a) Invasion cell number in SGC‐7901 cell line. (b) Invasion cell number in MGC‐803 cell line. *: *p* < .05, versus. NC group; #: *p* < .05, versus. Muscone group; $$: *p* < .01, versus. Muscone group

The number of invading SGC‐7901 cells was significantly lower in the muscone, miRNA, and muscone + miRNA groups than in the NC group (*p* < .05, respectively), and there were significant differences in the numbers of invading SGC‐7901 and MGC‐803 cells between the muscone group and the muscone + miRNA group (*p* < .05). However, the invasion rate of SGC‐7901 cells in the muscone + miRNA inhibitor group was significantly upregulated compared with the muscone group (*p* < .05). The data are shown in Figure 6.

### 3.6 Cell migration (wound healing assay)

**FIGURE 7:**
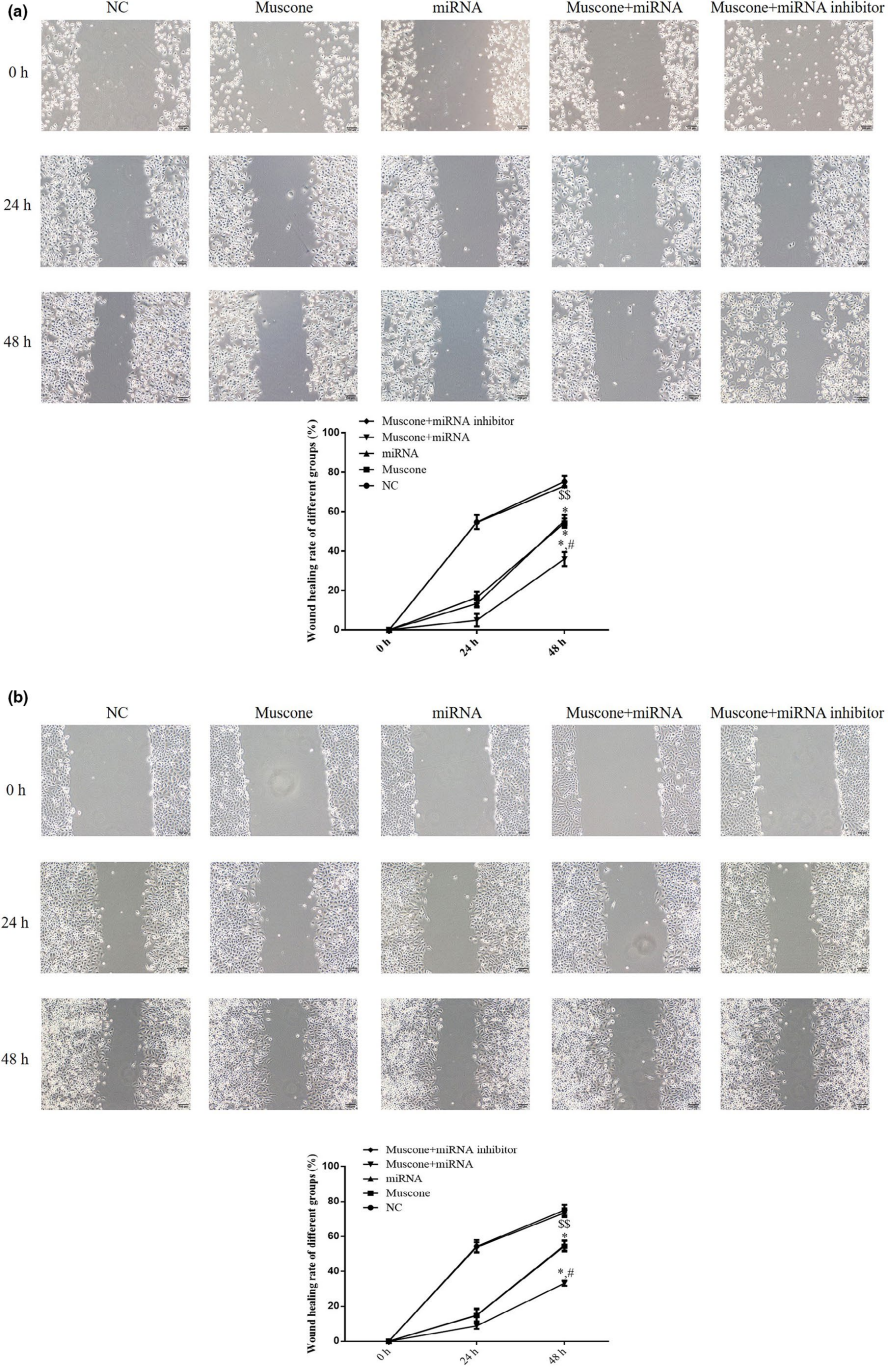
Wound healing rate by wound healing assay in difference groups (100×). (a) Wound healing rate in different SGC‐7901 cell line. (b) Wound healing rate in different MGC‐803 cell line. *: *p* < .05, versus. NC group; #: *p* < .05, versus. Muscone group; $$: *p* < .01, versus. Muscone group

To investigate the ability of cells to migrate, the wound healing rate was measured for the different groups using a wound healing assay. The wound healing rates of the muscone, miRNA, and muscone + miRNA groups were significantly suppressed compared with the NC group (*p* < .05), and there were significant differences between the muscone and muscone + miRNA groups (*p* < .05). However, the wound healing rate of the muscone + miRNA inhibitor group was significantly higher than that of the muscone group (*p* < .05). The respective data are shown in Figure 7.

### 3.7 Relative protein expression (Western blot assay)

**FIGURE 8:**
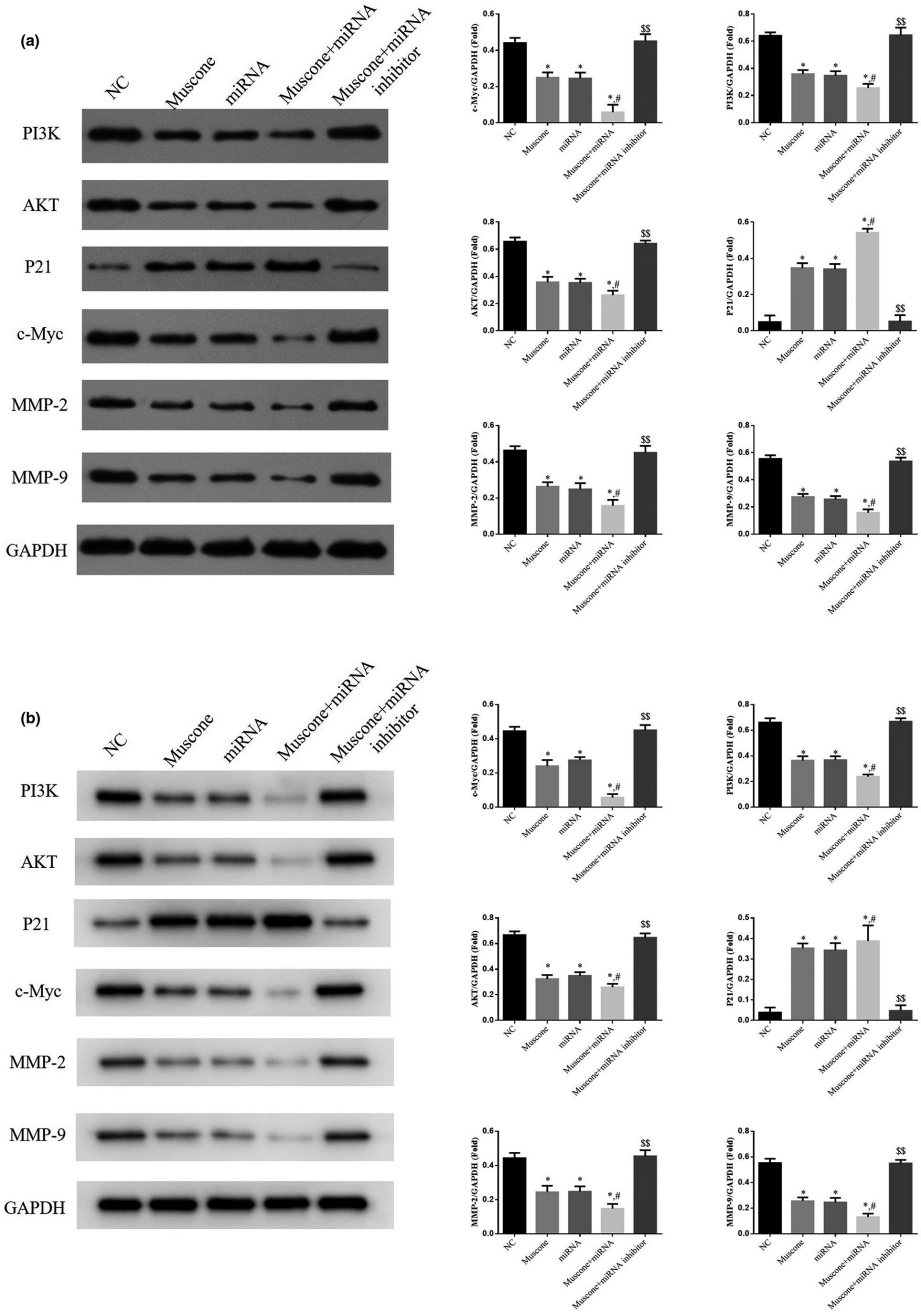
The relative proteins expressions. (a) Relative proteins expression in SGC‐7901 cell line. (b) Relative protein expression in MGC‐803 cell line. *: *p* < .05, versus. NC group; #: *p* < .05, versus. Muscone group; $$: *p* < .01, versus. Muscone group

The WB assay showed that in the muscone, miRNA, and muscone + miRNA groups, the expression of PI3K, AKT, c‐Myc, MMP‐2, and MMP‐9 proteins was significantly suppressed compared with the NC group, whereas P21 protein expression was significantly upregulated (*p* < .05). Furthermore, compared with the muscone group, expression of the proteins PI3K, AKT, c‐Myc, P21, MMP‐2, and MMP‐9 was significantly higher in the muscone + miRNA inhibitor group (*p* < .01 for all, Figure 8) but significantly lower in the muscone + miRNA group (*p* < .05, Figure 8).

### 3.8 Relative gene expression (RT‐qPCR assay)

**FIGURE 9:**
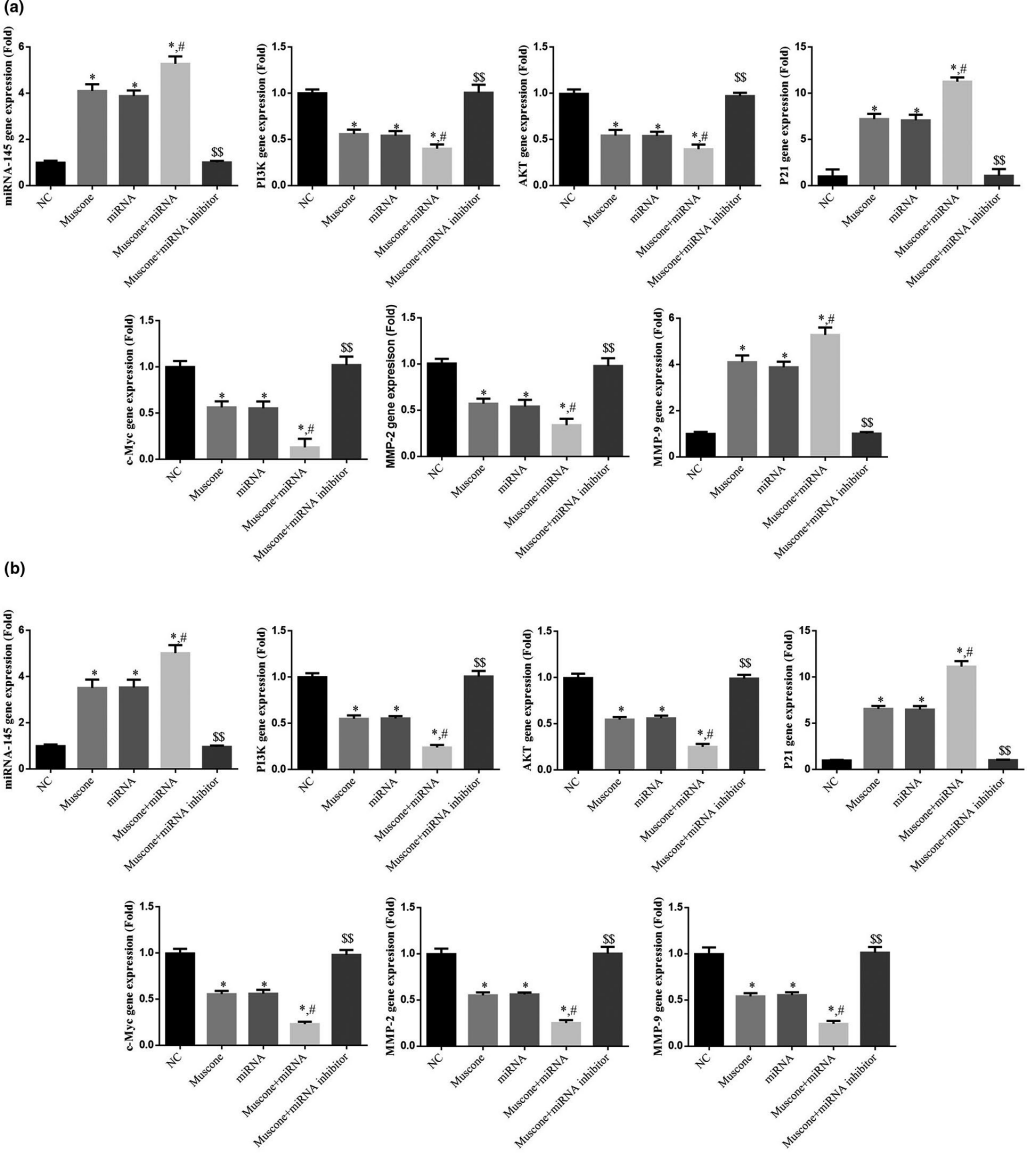
The relative mRNA expression. (a) Relative gene expression in SGC‐7901 cell line. (b) Relative gene expression in MGC‐803 cell line. *: *p* < .05, versus. NC group; #: *p* < .05, versus. Muscone group; $$: *p* < .01, versus. Muscone group

RT‐qPCR analysis revealed that in the muscone, miRNA, and muscone + miRNA groups, expression of the gene encoding miRNA‐145, as well as the *PI3K*, *AKT*, *c‐Myc*, *MMP‐2*, and *MMP‐9* genes, was significantly suppressed compared with the NC group, whereas expression of the *P21* gene was significantly upregulated (*p* < .05). Compared with the muscone group, expression of the gene encoding miRNA‐145, as well as the *PI3K*, *AKT*, *c‐Myc*, *P21*, *MMP‐2*, and *MMP‐9* genes, was significantly higher in the muscone + miRNA inhibitor group (*p* < .01 for all, Figure 9) but significantly lower in the muscone + miRNA group (*p* < .05 for all, Figure 9).

## 4 DISCUSSION

Gastric cancer is the fourth most common cancer in the world and the second most common cause of cancer death, with millions of cases every year (Zhang et al., 2015). The malignant proliferation, invasion, and metastasis of tumor cells are the most important problems in the development of tumors (Guo et al., 2014; Zheng et al., 2015). Muscone has inhibitory effects against breast and liver cancers in vitro (Bitsch et al., 2002; Qi et al., 2020; Wang et al., 2020). However, the strength and mechanism of action of muscone's anti‐cancer effects remain unclear. In recent years, many studies have reported that miRNA‐145 acts as a tumor suppressor in some cancers (Azmi et al., 2017; Chang et al., 2017; Wei & Li, 2017). In the present study, we found that muscone suppressed the biological activity of the gastric cancer cell line SGC‐7901, while muscone combined with miRNA‐145 had even stronger antitumor effects. However, adding miRNA‐145 inhibitor resulted in SGC‐7901 biological activity recovering despite the muscone treatment. Based on these results, we infer that the inhibitory effects of muscone against gastric cancer cells may be correlated with stimulation of miRNA‐145 activity.

The PI3K/AKT pathway is involved in many important biological processes. It plays a key role in inhibiting cell apoptosis, promoting proliferation, and stimulating invasion and migration by affecting the activation state of many downstream effectors (Liu et al., 2017; Xu et al., 2017; Zhang et al., 2016; Zheng et al., 2014). Previous studies have reported that miRNA‐145 suppresses cancer cell activity by regulating the PI3K/AKT pathway (Liu, Gao, et al., 2017; Zhang, Yan, et al., 2015). Previous studies (Liu, Gao, et al., 2017; Zhang, Yan, et al., 2015) have also shown that miRNA‐145 is downregulated in cancer cells and that miRNA‐145 overexpression significantly suppresses cellular biological activity via regulation of the PI3K/AKT pathway. In this study, expression of the proteins PI3K and AKT was suppressed in the muscone and muscone + miRNA groups; however, expression of the proteins PI3K and AKT in the presence of muscone was restored by miRNA‐145 inhibitor transfection. These results suggest that muscone may inhibit the PI3K/AKT pathway in vitro via regulation of miRNA‐145.

c‐Myc and P21 play important roles in regulating downstream gene targets of the PI3K/AKT pathway (Xu & Dang, 2017; Yang et al., 2017), as well as serving key functions in cell proliferation and apoptosis (Cao et al., 2018; Lv et al., 2017). In the muscone, miRNA, and muscone + miRNA groups in this study, cell proliferation was inhibited and cell apoptosis rate was increased, with most cells remaining in the G1 phase of the cell cycle. However, miRNA‐145 inhibition resulted in increased cell proliferation and decreased cell apoptosis rate. We infer that the ability of muscone to suppress cell proliferation and increase cell apoptosis in vitro may be related to c‐Myc and P21 expression.

Matrix metalloproteinases (MMPs) are important factors in the invasiveness of many types of cancers, such as colon cancer and breast cancer. MMP‐2 and MMP‐9 are two important members of the MMP family, and the genes encoding them are downstream targets of the PI3K/AKT pathway (Jiang et al., 2018; Lan et al., 2017). Furthermore, overexpression of MMP‐2 and MMP‐9 is closely correlated with tumor invasion and migration (Shi et al., 2017; Wang et al., 2018). In this study, the expression of MMP‐2 and MMP‐9 proteins was suppressed by muscone, miRNA, and muscone + miRNA; however, in the muscone + miRNA inhibitor group, the expression of MMP‐2 and MMP‐9 proteins recovered. Based on these results, we infer that muscone may inhibit invasion and migration of SGC‐7901 and MGC‐803 cells by regulating MMP‐2 and MMP‐9 protein expression via the PI3K/AKT pathway.

In conclusion, these results suggest that muscone exerts antitumor effects in vitro against the gastric cancer cell line SGC‐7901 via a pathway involving PI3K, AKT, c‐Myc, MMP‐2, and MMP‐9.
